# Antidepressant-like Effect of a Chalcone Compound, DHIPC and Its Possible Mechanism

**Published:** 2018

**Authors:** Dong-Hai Zhao, Yan-Chun Wang, Lian-Wen Zheng, Bing-Yu Liu, Li-Ping Guan

**Affiliations:** a *Department of Pathology, Jilin Medical University, Jilin, Jilin Province 132013, P. R. China. *; b *Department of Obstetrics and Gynecology, Second hospital, Jilin University, Changchun 130041, P. R. China.*; c *Food and Pharmacy College, Zhejiang Ocean University, Zhoushan, Zhejiang, 316022, P. R. China.*

**Keywords:** Chalcone, DHIPC, Antidepressant-like activity, Forced swimming test, Tail suspension test, Neurotransmitters

## Abstract

DHIPC (2,4-dichloro-2´-hydroxyl-4´,6´-diisoprenyloxychalcone) is a new chalcone compound. In this study, its antidepressant-like activity of compound DHIPC was evaluated by the forced swimming test and the tail suspension test in mice. The results showed that DHIPC significantly reduced the immobility time for 2 h after treatment through the oral administration at dose of 10, 20, and 30 mg/kg in the forced swimming test and the tail suspension test, indicating a significant antidepressant-like effect. The maximal effect was obtained at 30 mg/kg, which is similar to the positive control fluoxetine. The main monoamine neurotransmitters and their metabolites in rat brain were also simultaneously determined. It was found that DHIPC significantly increased the concentrations of the main neurotransmitters serotonin and noradrenalin, and also significantly increased 5-hydroxyindoleacetic acid contents in hippocampus, hypothalamus, and cortex in brain part. So, the probable mechanism of action of DHIPC is thought to be related to increase in serotonin and noradrenalin in the brain.

## Introduction

**Figure 1:**
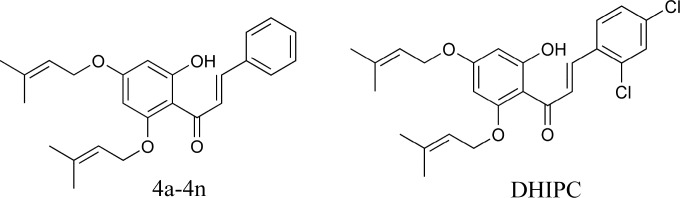
Structure of compounds 4a-4n and DHIPC

Depression is one of the most prevalent psychopathologies. Symptoms of depression include lowered mood and reduced interest and pleasure. It is a chronic and disabling mental illness that causes high morbidity and mortality (1). According to world health organization (WHO) prediction, depression will be the second most common disease in 2020 (2, 3). Approximately two-thirds of the depressed patients respond to the currently available treatments but the magnitude of improvement is still disappointing. Although there are many effective antidepressants available today, the current armentarium of therapy is often inadequate with unsatisfactory results in about one-third of all subjects treated (4-6). Therefore, the discovery of new antidepressant drugs with fewer side-effects and better efficacy is necessary. The subclasses of flavonoids are the phenolic α, β-unsaturated ketones chalcones, which contain a 1,3-diphenyl-2-en-1-one core. Chalcones are one of the major classes of naturally occurring compounds highly widespread in n vegetables, fruits, spices, tea, and soy-based foodstuff. They have been subject of great interest for their remarkable pharmacological activities (7, 8). In recent years, the antidepressant effects of flavonoids and chalcones have caused widespread interest and have been widely studied (9-14). In our research team search for the new compounds with the antidepressant-like activity, we synthesized 14 new 2′-hydroxy-4′,6′-diisoprenyloxychalcone derivatives and evaluated for their antidepressant-like activity through injected intraperitoneally in mice. The pharmacological results showed that compound DHIPC(2,4-dichloro-2´-hydroxyl-4´,6´- diisoprenyloxychalcone) (Figure 1) was found to be the most antidepressant -like effect, and also it significantly reduced the duration of immobility time at 10 mg/kg dose level when compared to the control (*p *< 0.001) in the forced swimming test and the tail suspension test (15).

In the present study, we synthesized compound DHIPC and evaluated its antidepressant-like activity in mice by two classic animal behavior despair tests; the forced swimming test and the tail suspension test through injected oral administration. The main monoamine neurotransmitters and their metabolites were simultaneously determined by HPLC–ECD to investigate the probable mechanism of action of DHIPC.

## Experimental

*Drugs, agents and animals*

DHIPC was synthesized in our laboratory (Zhejiang Ocean, Zhoushan, China) (15). Its structure was characterized using IR, ^1^H-NMR, ^13^C-NMR, MS and elemental analysis techniques. A positive control: fluoxetine-HCl (Sigma, Shanghai, purity > 99%) was included. Reference standards for simultaneous determination of noradrenaline, serotonin, dopamine, and 5-hydroxyindoleacetic acid were all purchased from Sigma (Shanghai, purity > 99%). The major chemicals were purchased from Aldrich Chemical Corporation (Shanghai, China). All mice were obtained from the Laboratory of Animal Research, College of Pharmacy, Zhejiang Academy of Medical Sciences.

**Table 1:** Evaluation of the antidepressant-like activity of DHIPC in the forced swim test

**Compounds**	**Dosage** **(mg/kg)**	**Antidepressant activity** a	
		**Duration of immobility(s)**	**DID (%)** b
DHIPC	10	89.5 ± 9.8*	30.46
	20	68.9 ± 11.3**	46.46
	30	53.3 ± 11.3**	58.59
fluoxetine	20	55.3 ± 8.1***	57.03
Control	**—**	128.7 ± 12.7	**—**

a DHIPC and fluoxetine were administered intraperitoneally. Values are the mean ± SEM (*n *= 8).

* Significantly different compared with control (^*^p < 0.05,

** *p *< 0.01

*** *p *< 0.001

b % DID: percentage decrease in immobility duration.

**Table 2:** Effects of DHIPC treatment on neurotransmitter concentrations the forced swim test

**Region**	**Group**	**serotonin**	**noradrenaline**	**5-hydroxyindoleacetic acid**	**dopamine**
hypothalamus	stress vehicle	245.2 ± 11.4^z^	193.7 ± 10.2^z^	355.1 ± 24.6^z^	209.3 ± 13.4
	DHIPC	467.7 ± 48.9^a^	297.7 ± 11.6^b^, ^z^	512.2 ± 17.2^a^	199.7 ± 10.6
	fluoxetine	475.2 ± 51.4^a^	305.3 ± 47.1^b^	585.6 ± 26.5^a^, ^z^	186.9 ± 9.3
	vehicle control	605.4 ± 12.2^a^	486.4 ± 57.4^a^	914.2 ± 35.1^a^	192.4 ± 34.3
hippocampus	stress vehicle	267.4 ± 11.7^y^	107.3 ± 9.3^z^	295.4 ± 19.6^y^	40.3 ± 10.6
	DHIPC	334.5 ± 9.6b, z	173.3 ± 10.8^z^	389.6 ± 25.7^b^, ^z^	42.6 ± 13.3
	fluoxetine	357.1 ± 14.2^b^, ^z^	184.1 ± 12.6^b^, ^z^	357.7 ± 17.8^b^, ^y^	39.9 ± 7.9
	vehicle control	501.2 ± 46.1^a^	245.3 ± 14.4^b^	809.7 ± 20.5^a^	50.3 ± 12.6^z^
cortex	stress vehicle	204.2 ± 28.5^z^	154.1 ± 13.1^z^	177.5 ± 13.8^y^	1013.4 ± 65.1^z^
	DHIPC	451.7 ± 22.7^b^	196.2 ± 9.8^c^	186.3 ± 15.6^y^	1103.4 ± 73.0^y^
	fluoxetine	479.2 ± 40.3^b^, ^z^	201.1 ± 8.4^b^	197.7 ± 12.4^z^	1109.2 ± 68.1^z^
	vehicle control	645.7 ± 60.2^a^	223.5 ± 20.8^b^	299.2 ± 15.7^b^	1810 ± 98^c^

The dose of DHIPC and fluoxetine was 30 mg/kg. Neurotransmitter concentrations were expressed as ng/g per brain region wet weight. Data expressed as mean ± SEM (*n = *10). Statistical analysis of data was carried out by one-way analysis of variance followed by the Turkey’s test.

a *p *< 0.001,

b *p *< 0.01,

c *p *< 0.05 *vs*. Stress vehicle,

x *p *< 0.001,

y *p *< 0.01,

z *p *< 0.05 *vs. *vehicle control.

**Table 3:** Effects of DHIPC treatment on neurotransmitter concentrations in the tail suspension test

**Region**	**Group**	**serotonin**	**noradrenaline**	**5-hydroxyindoleacetic acid**	**dopamine**
hypothalamus	stress vehicle	255.1 ± 22.4^x^	55.8 ± 6.2^z^	415.2 ± 38	109.1 ± 10.8^z^
	DHIPC	385.1 ± 23.6^b^	99.7 ± 7.6^c^,^z^	843.4 ± 65^a^,^z^	106.4 ± 9.3
	fluoxetine	397.1 ± 20.4^b^	101.5 ± 6.4^b^,^z^	866.5 ± 48.2^a^,^z^	117.2 ± 13.2
	vehicle control	732.4 ± 50.8^a^	199.7 ± 11.2^a^	901.1 ± 92 x	188.3 ± 12.4^c^
hippocampus	stress vehicle	132.4 ± 10.5^y^	103.1 ± 5.7^y^	1313.5 ± 73.3^y^	76.2 ± 5.4^z^
	DHIPC	242.4 ± 10.0^b^,^y^	163.9 ± 8.8^b^,^z^	1583.6 ± 30.7^b^,^z^	78.4 ± 5.3
	fluoxetine	250.8 ± 9.1^b^,^y^	194.2 ± 6.8^a^,^z^	1521.4 ± 42.7^b^	80.6 ± 4.8
	vehicle control	682.1 ± 40.8^a^	291.6 ± 11.1^a^	1129.2 ± 59.7	93.6 ± 9.1^c^
cortex	stress vehicle	342.4 ± 19.5^y^	96.5 ± 9.9^x^	190.2 ± 13.4^x^	1381.3 ± 59.1^y^
	DHIPC	498.5 ± 25.4^b^	125.4 ± 6.9^c^	149.1 ± 7.6^z^	1130.4 ± 63.3^z^
	fluoxetine	554.5 ± 44.3^a^	146.8 ± 5.4^b^	150.2 ± 10.4^z^	1248.1 ± 99.1^z^
	vehicle control	673.5 ± 62.3^a^	241.7 ± 9.8^a^	339.4 ± 11.7^a^	2134.1 ± 142.1^b^

The dose of DHIPC and fluoxetine was 30 mg/kg. Neurotransmitter concentrations were expressed as ng/g per brain region wet weight. Data expressed as mean ± SEM (*n *= 10). Statistical analysis of data was carried out by one-way analysis of variance followed by the Turkey’s test.

a *p *< 0.001,

b *p *< 0.01,

c *p *< 0.05 *vs.* Stress vehicle,

x *p *< 0.001,

y *p *< 0.01,

z *p *< 0.05 *vs. *vehicle control.

**Figure 2:**
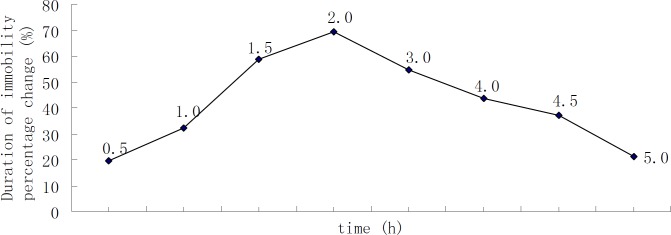
Time-course of DHIPC in the forced swim test (the number of animals at each point was 10).

**Figure 3:**
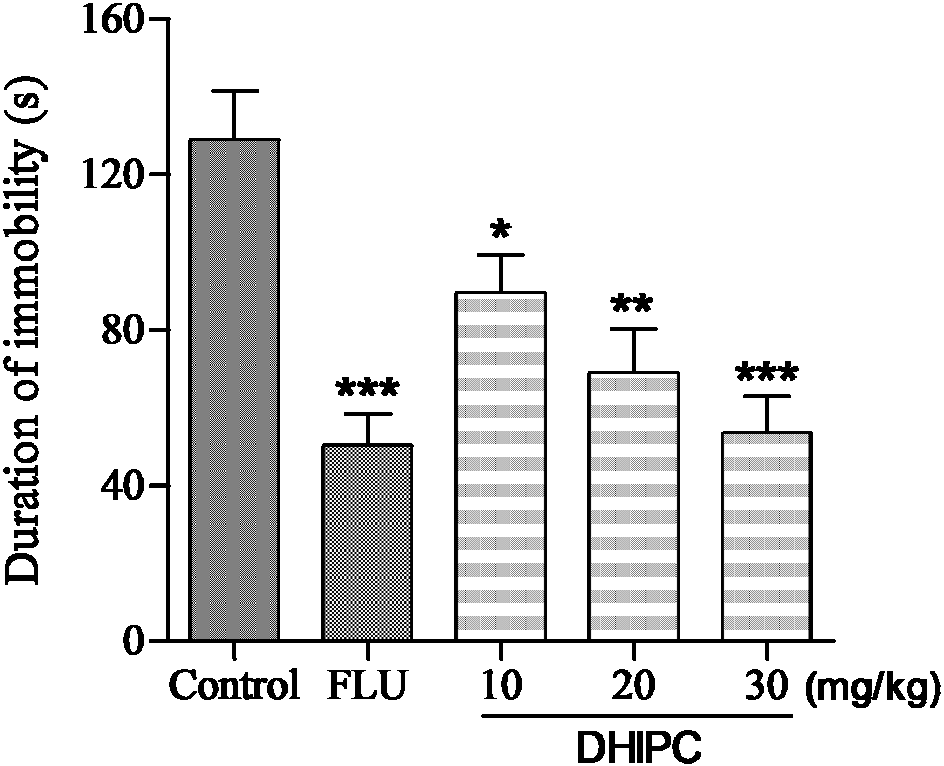
Effect of DHIPC on immobility time in the forced swim test in mice. Data are expressed as the mean ± SEM (*n* = 8). Symbol (*, ** or ***) indicates statistically signiﬁcance in comparison to vehicle at *p *< 0.05, *p *< 0.01, *p *< 0.001

**Figure 4:**
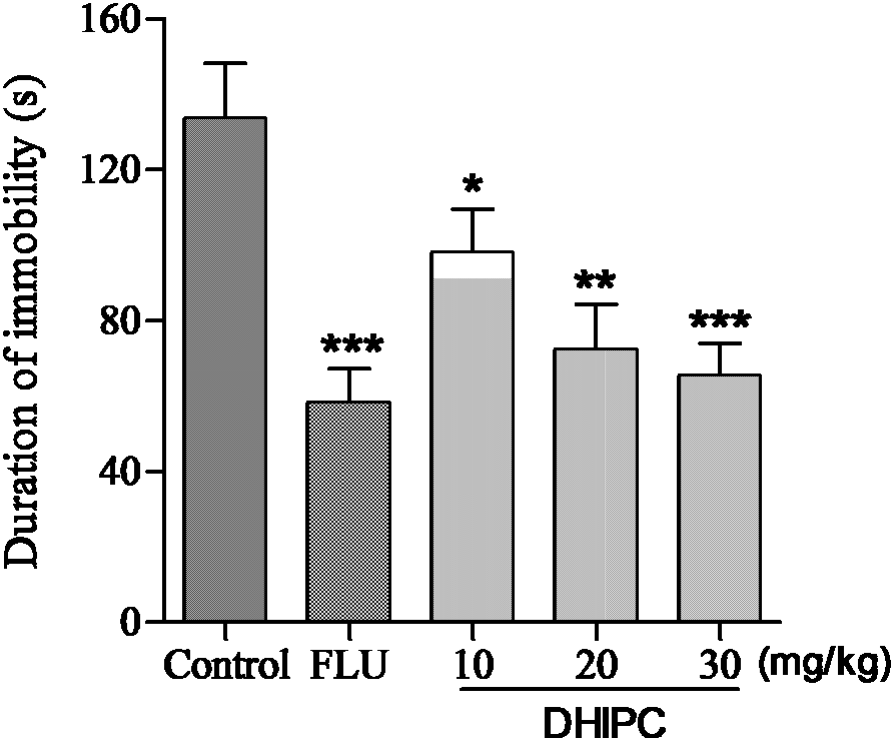
Effect of DHIPC on immobility time in tail suspension test in mice. Data are expressed as the mean ± SEM (*n* = 8). Symbol (*, ** or ***) indicates statistically signiﬁcance in comparison to vehicle at* p *< 0.05, *p *< 0.01, *p *< 0.001

*Drug treatment*

DHIPC was dissolved in 0.5% methylcellulose cellulose sodium and administered per-oral injection at doses of 10, 20, and 30 mg/kg, which was designed after the pretest. Fluoxetine was dissolved in sodium chloride and was also administered acute per-oral injection at dose of 20 mg/kg. The volume of administration for vehicle and drug solutions was 0.2 mL/20 g of mice, and other drugs were dissolved in 0.9% isotonic saline solution immediately before use. The vehicle or test drugs were administered 2 h before the test session. The procedures in this study were performed in accordance with the National Institute of Health Guide for the Care and Use of Laboratory Animals and approved by the Ethics Committee of our Institution. All efforts were made to minimize animals suffering and to reduce the number of animals used in the experiments.

*Forced swimming test*

Male ICR mice (20 ± 2 g) were used in the forced swim test under standard conditions with free access to food and water. The mice were randomly divided into five groups (8 mice per group were used) for the forced swim test. We used 40 male mice. On the test day, the mice were dropped once a time into a plexiglass cylinder (height 25 cm, diameter 10 cm) containing 10 cm of water at 20 ± 2 °C. In this day, the mice were assigned into different groups (*n* = 8 for each group). The vehicle or test drugs were administered 2 h before a test session oral injection. Then, the mice were dropped individually into the plexiglass cylinder and left in the water for 6 min. After the first 2 min of the initial vigorous struggling, the animals were immobile. The duration of immobility was recorded during the last 4 min of the 6 min test. All test swim sessions were recorded by a video camera positioned directly above the cylinder. Two competent observers, who were unaware of the treatment each mouse had received, scored the videotapes. Immobility period was regarded as the time spent by the mouse floating in the water without struggling and making only those movements necessary to keep its head above the water. Following swimming sessions, they were then towel dried and returned to their housing condition. The animals were used only once in this test. All the forced swim tests were performed between 11:00 am and 14:00 pm (16, 17).

*Tail suspension test*

Local breed, male ICR mice (20 ± 2 g) were used in the forced swim test under standard conditions with free access to food and water. Mice were randomly divided into five groups (8 mice per group were used) for the tail suspension test*.* We used 40 male mice. Briefly, the vehicle or test drugs were administered 2 h before a test session oral injection. Then, mice were individually suspended by tail with clamp (2 cm from the tip of the end) in a box (25 × 25 × 30 cm) with the head 5 cm to the bottom. Testing was carried out in a darkened room with minimal background noise. All animals were suspended for total 6 min, and the duration of immobility was observed and measured during the final 4-min interval of the test. All test sessions were recorded by a video camera positioned directly above the box. Two competent observers blind to treatment scored the videotapes. Mice consider immobile only when they hung passively and completely motionless. The animals were used only once in this test. All tail suspension tests were performed between 12:00 am and 15:00 pm (18, 19). 

*Time-course of oral administration on* *the forced swim test*

The time-course effect of DHIPC in the forced swim test was determined. A 30 mg/kg dose of DHIPC suspension in a mixture containing 0.5% methylcellulose cellulose sodium was injected per-oral in the mice. The mice were divided into 7 groups. Subsequently, the animals were subjected to the forced swim test at various times: 0.5, 1.0, 1.5, 2.0, 3.0, 4.0, and 5.0 h. 

*The sample preparation*

Quantification of monoamines was performed according to the method described in Chen *et al.* (20) with some modifications. The doses 30 mg/kg of DHIPC were tested the effect on neurotransmitter concentrations in the brain of rat. Rats were randomly divided into four groups (10 mice per group were used); we used 40 male mice. DHIPC (30 mg/kg), fluoxetine (30 mg/kg), vehicle control, stress vehicle were given orally daily for 7 days. In the last day, the drugs were given 1 h prior to the test. At the end of the experiment, the rats were immediately sacrificed by cervical dislocation; the brain tissue was quickly removed, and rapidly frozen and stored at −80 °C until they were processed for biochemical estimations.

*HPLC condition and test*

Following decapitation, the brains were rapidly removed, dissected on an ice-chilled glass plate and subsequently prefrontal hypothalamus, cortex and hippocampus were isolated. The tissues were weighed, sonicated in 0.1M NaH_2_PO_4_ aqueous solution including 0.85 mM OSA, 0.5 mM EDTA·Na_2_ (ethylenediamine tetraacetic acid disodium) and centrifuged (12000×*g* for 30 min). Then, noradrenaline, serotonin, dopamine, and 5-hydroxyindoleacetic acid were assayed by HPLC-ECD. The HPLC system consisted of a microbore reverse-phase column (Shimadzu LC-10ATVP HPLC system, Shimadzu L-ECD-6A electrochemical detector, N2000 HPLC workstation software, Hypersil ODS C18 Column 4.6 × 150 mm 5 μM Thermo, USA). The mobile phase consisted of 0.1M NaH_2_PO_4_ aqueous solution including 0.85 mM OSA, 0.5 mM EDTA·Na_2_ and 11% methanol adjusted to pH 3.4 with phosphate acid and filtered through 0.45 μM pore size filter. External standard curves were used to quantify the amounts of noradrenaline, serotonin, dopamine and 5-hydroxyindoleacetic acid in each sample calculated by area under curve. The volume of injection was 20 μL. The detection limit of the assay was 20 pg/g sample. The filtrate sample was used for quantification of serotonin (*Y *= 0.0529 X+0.0016,* R *= 0.9989), noradrenaline (*Y *= 0.0357 X+0.0031,* R *= 0.9991), dopamine (*Y *= 0.0628 X+0.00417,* R *= 0.9976), and 5-hydroxyindoleacetic acid (*Y *= 0.0481 X+0.0053,* R *= 0.9988) by HPLC coupled with electro-chemical detection in all brain regions. 

*Statistical analysis*

The results are expressed as mean ± SEM n representing the number of animals. The statistical tests used were one-way analysis of variance (ANOVA) and Tukey›s test for comparison between all treatment groups using GraphPad Prism program (GraphPad Software, Inc., San Diego, USA). A *p*-value of less than 0.05 was considered statistically significant. 

## Results and Discussion

*Forced swimming test through injected per-oral*

Firstly, the time-course effect of DHIPC in the forced swimming test was determined. The peak effect of an oral dose of 30 mg/kg DHIPC was exhibited between 0.5 and 5 h after administration (Figure 2). The peak of protection was observed 2 h after the* per-oral* injection. The time-to-peak effect was maximal at 2 h. DHIPC exhibited the antidepressant-like activity and promoted a significant decrease in the immobility time.

*Effect on immobility periods of DHIPC* *through injected per-oral*

The antidepressant-like activity of DHIPC and fluoxetine on the immobility time in the forced swim test are presented in Figure 3. There was a significant change in immobility time after 2 h treatment with DHIPC at doses of 10, 20, and 30 mg/kg, which exhibited a significant decrease in the immobility times after per-oral administration, indicating a significant antidepressant-like effect. There was a significant treatment effect for dose in immobility time. The maximal effect was obtained at 30 mg/kg, which is similar to the positive control fluoxetine, and in particular led to significant reductions in the duration of the immobility time compared to the control group with *p *< 0.001. To better understand the antidepressant effects of DHIPC and fluoxetine, we calculated the percentage decrease in immobility duration (% DID) using the formula % DID = [(*Y *- *X*)/*Y*] × 100, where *Y* is the duration of immobility (s) in the control group, and *X* is the duration of immobility (s) in the test group. The duration of immobility in the forced swim test was reduced and the % DID increased by three doses of DHIPC in the forced swim test. As shown in Table 1, the majority of DHIPC reduced the duration of immobility and gave high % DID values. In comparison to control group receiving the vehicle, the inhibitions were 30.46%, 46.46%, and 58.59%, respectively. Among these, the most promising antidepressant DHIPC (% DID = 58.59) reduced the immobility duration at 30 mg/kg dose level in the forced swim test, which is similar to fluoxetine (% DID = 57.03%).

The same effects were observed for three doses of DHIPC in the tail suspension test (Figure 4), the decrease in the immobility time displayed similarity to that seen in the forced swim test (as duration of immobility (s): control: 133.7 ± 14.7, DHIPC: 10 mg/kg = 98.2 ± 11.5, 20 mg/kg = 72.4 ± 12.1, 30 mg/kg = 65.7 ± 8.4, fluoxetine = 58.4 ± 9.0), and the inhibitions were 26.55%, 45.85%, and 50.86 %, respectively, indicating a significant antidepressant-like effect. The maximal effect was obtained at 30 mg/kg, which is similar to the positive control fluoxetine, showing the most antidepressant-like activity. These ratios results suggested that DHIPC were adequately absorbed in mice after oral administration. These results also indicate that DHIPC has a significant antidepressant activity in mice during the forced swim test and tail suspension test. Furthermore, DHIPC exhibited the antidepressant-like effect in a dose-dependent manner. The forced swim test and tail suspension test detects the anti-immobility effects of a wide array of antidepressants, involving monoamine oxidase inhibitors, tricyclic antidepressants, selective serotonin reuptake inhibitors, and the atypical antidepressants. Thus, the antidepressant effects of DHIPC may be include one of the mechanisms of the established agents as described above (21, 22).

*Probable mechanisms of antidepressant-like activity of DHIPC*

The levels of monoamine neurotransmitters and their metabolites detected in rat brain are summarized in Tables 2 and 3. Interestingly, in this study, we found that DHIPC significantly increased noradrenaline and serotonin concentrations at the highest doses in rat hypothalamus, hippocampus and cortex in both the forced swim test and tail suspension test, similar to the positive control drug fluoxetine. No significant changes in dopamine levels in all measured brain regions observed following DHIPC administration compared with the stress vehicle group, while there are also significant changes in 5-hydroxyindoleacetic acid levels in some brain parts, especially in hypothalamus, indicating a reduced serotonin metabolism. Our findings suggest that the antidepressant-like activity of DHIPC is likely mediated through increased noradrenaline and serotonin levels in the central nervous system. Some studies have also shown the adaptogenic effect of the plant extract via normalization of the various stress parameters and monoaminergic levels, which may provide a clue that the extract is bringing their possible antidepressant effect through restoration of normal monoaminergic neurotransmitters (23, 24). Dysregulation of the central nervous system neurotransmitters noradrenaline, serotonin, and dopamine are thought to play a role in the pathogenesis of depression. 

The majority of studies have focused on the noradrenaline and serotonin systems. A metabolic disorder of monoamine neurotransmitters is believed to be the main biochemical cause of depression, and increasing the levels of monoamine neurotransmitters in the central nervous system can alleviate the symptoms of depression (25-27). Reduced concentrations of 5-hydroxyindol-eacetic acid have been observed in the brains of patients and animals experiencing stress and depression, indicating a dysfunction of the serotonergic system (29, 30).

## Conclusions

In conclusion, DHIPC evaluated the antidepressant-like effect in the forced swim test and tail suspension test through the oral administration. 

The results displayed that DHIPC significantly reduced the immobility time in the forced swim test and tail suspension test in mice at dose of 10, 20, and 30 mg/kg in mice and showed the antidepressant-like activity. In addition, DHIPC significantly increased the concentrations of serotonin, noradrenalin, and 5-hydroxyindoleacetic acid contents in hippocampus, hypothalamus, and cortex in brain part. So, the probable mechanism of action of DHIPC is thought to be related to increase in serotonin and noradrenalin in the brain.
